# Signatures of selection are present in the genome of two close autochthonous cattle breeds raised in the North of Italy and mainly distinguished for their coat colours

**DOI:** 10.1111/jbg.12659

**Published:** 2021-11-28

**Authors:** Francesca Bertolini, Giulia Moscatelli, Giuseppina Schiavo, Samuele Bovo, Anisa Ribani, Mohamad Ballan, Massimo Bonacini, Marco Prandi, Stefania Dall’Olio, Luca Fontanesi

**Affiliations:** ^1^ National Institute of Aquatic Resources Technical University of Denmark Kongens Lyngby Denmark; ^2^ Division of Animal Sciences Department of Agricultural and Food Sciences University of Bologna Bologna Italy; ^3^ Associazione Nazionale Allevatori Bovini di Razza Reggiana (ANABORARE) Reggio Emilia Italy

**Keywords:** *Bos taurus*, coat colour, genetic resource, genome, population genomics

## Abstract

Autochthonous cattle breeds are genetic resources that, in many cases, have been fixed for inheritable exterior phenotypes useful to understand the genetic mechanisms affecting these breed‐specific traits. Reggiana and Modenese are two closely related autochthonous cattle breeds mainly raised in the production area of the well‐known Protected Designation of Origin Parmigiano‐Reggiano cheese, in the North of Italy. These breeds can be mainly distinguished for their standard coat colour: solid red in Reggiana and solid white with pale shades of grey in Modenese. In this study we genotyped with the GeneSeek GGP Bovine 150k single nucleotide polymorphism (SNP) chip almost half of the extant cattle populations of Reggiana (*n* = 1109 and Modenese (*n* = 326) and used genome‐wide information in comparative F_ST_ analyses to detect signatures of selection that diverge between these two autochthonous breeds. The two breeds could be clearly distinguished using multidimensional scaling plots and admixture analysis. Considering the top 0.0005% F_ST_ values, a total of 64 markers were detected in the single‐marker analysis. The top F_ST_ value was detected for the *melanocortin 1 receptor* (*MC1R*) gene mutation, which determines the red coat colour of the Reggiana breed. Another coat colour gene, *agouti signalling protein* (*ASIP*), emerged amongst this list of top SNPs. These results were also confirmed with the window‐based analyses, which included 0.5‐Mb or 1‐Mb genome regions. As variability affecting *ASIP* has been associated with white coat colour in sheep and goats, these results highlighted this gene as a strong candidate affecting coat colour in Modenese breed. This study demonstrates how population genomic approaches designed to take advantage from the diversity between local genetic resources could provide interesting hints to explain exterior traits not yet completely investigated in cattle.

## 1 INTRODUCTION

Autochthonous cattle breeds constitute important genetic resources, in many cases unexploited or poorly characterized. Many local breeds have been developed by the combined action of several factors and events mainly driven by the recent interplay between economic, social and environmental conditions that contributed to define their genetic history (e.g. Felius et al., 2014). Directional selection, that finally shaped the current genetic pools, fixed or almost fixed inheritable exterior phenotypes that could be considered breed‐specific traits useful to distinguish different breeds. One of the main exterior phenotypes that characterize different breeds is coat colour.

Two autochthonous cattle breeds, Reggiana and Modenese, are raised mainly in the production area of the well‐known Protected Designation of Origin (PDO) Parmigiano‐Reggiano cheese, in the North of Italy. Reggiana and Modenese are historically considered the ancestral cattle populations from that this cheese has been originated. Their names derive from the two geographically close provinces of Reggio Emilia and Modena, located in the Emilia‐Romagna region where they have been mainly originated and where most of the farms raising Reggiana and Modenese cattle are now localized. The Herd Book of these two breeds were officially recognized in 1962 (Reggiana) and in 1957 (Modenese). In recent history, between the 1980’ and in first years of the 2000, both breeds have experienced a progressing decline of the population size that reached a minimum of approximately 500 Reggiana heads and 260 Modenese heads. Since then, the population size has been increasing. The recovery of these two breeds can be mainly attributed to the development of two mono‐breed branded Parmigiano‐Reggiano cheeses that can be produced from milk of only Reggiana cows or from milk of only Modenese cows. The market prize of these niche products is higher than the undifferentiated Parmigiano‐Reggiano cheese obtained by cosmopolitan breeds, hereby compensating the reduced milk yield that characterize these two breeds (Fontanesi, 2009; Gandini et al., 2007; Petrera et al., 2016; Russo et al., 2007). In 2020, Reggiana and Modenese accounted a total of approximately 2800 cows (raised in approximately 100 farms) and 500 cows (raised in approximately 60 farms), respectively.

**FIGURE 1:**
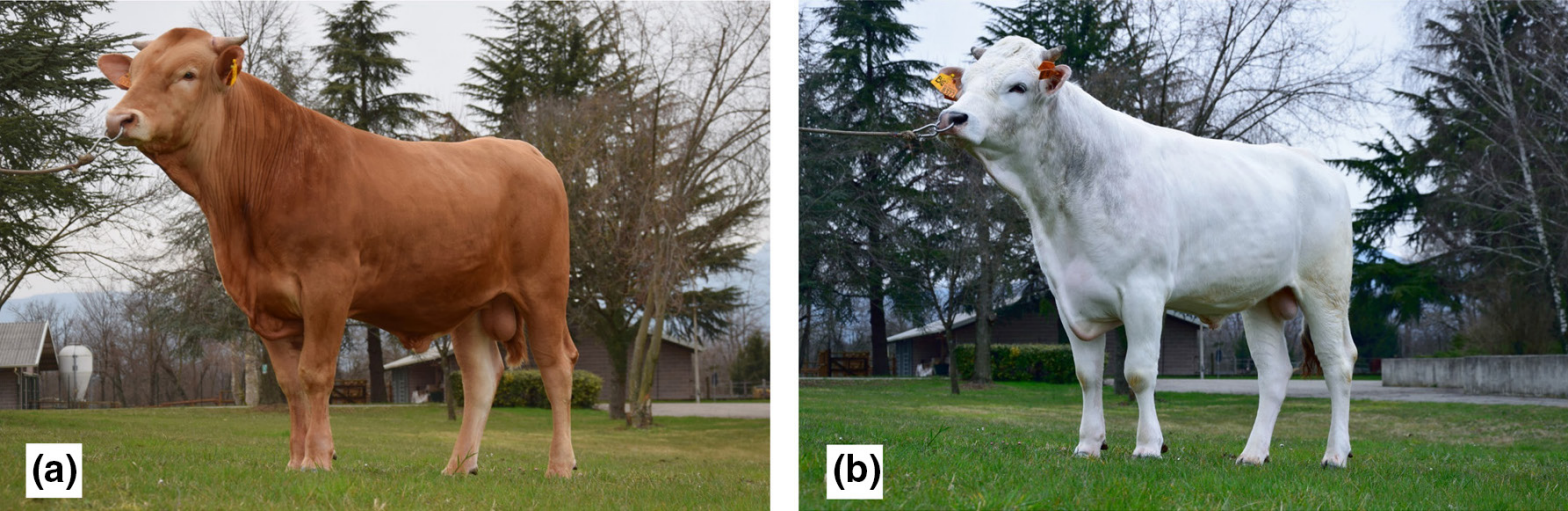
Pictures of Reggiana (a) and Modenese (b) sires [Colour figure can be viewed at wileyonlinelibrary.com]

Reggiana and Modenese can be distinguished according to their standard coat colour and muzzle colour (Figure 1): a classical red coat colour, indicated with the term “fromentino,” over the whole body, with pink or pale muzzle colour are the main pigmentation features of the Reggiana cattle; white coat colour of the body with some pale grey shades and a black muzzle with a depigmented inverted “V” are the main characteristics of Modenese cattle, also known as Bianca Val Padana (Bianca = White) that is the second name of this breed, which derives from its coat colour.

A few studies that investigated DNA markers in candidate genes and at the genome‐wide level were carried out in both breeds, using relatively small sample sizes. Here, candidate gene markers were used in association studies with production traits in Reggiana sires (Fontanesi et al., 2015) and to compare the frequency of relevant alleles affecting milk production traits amongst breeds, which included Reggiana and Modenese cattle (Fontanesi et al., 2007; Scotti et al., 2010). Polymorphisms in three coat colour‐affecting genes have been analysed in these breeds to identify markers useful to authenticate the breed origin of the mono‐breed Parmigiano‐Reggiano cheeses (Russo et al., 2007; Fontanesi et al., 2010; Fontanesi et al., 2012, 2010). Here, the melanocortin 1 receptor (*MC1R*) allele caused by a frameshift mutation (allele e) has been indicated to determine the red coat colour in Reggiana cattle (Klungland et al., 1995; Russo et al., 2007 **)**. A genome‐wide association study for exterior traits has been recently carried out in Reggiana (Bovo et al., 2021) and genome‐wide analyses of signature of selection and population genomic parameters have been carried out in Reggiana against a few other cosmopolitan and local cattle breeds (Bertolini et al., 2015,2018,2020; Mastrangelo, Ciani, et al., 2018; Mastrangelo et al., 2016). Modenese breed was analysed using single nucleotide polymorphism (SNP) information in a comparative analysis with Holstein and Maremmana breeds (Catillo et al., 2018). Genetic relationships amongst Italian cattle breeds that included SNP chip data from few Reggiana and Modenese cattle indicated that the two breeds are closely related compared to several other breeds (Mastrangelo, Sardina, et al., 2018).

Considering the limited information that is available in terms of genomic differences between these two geographically close breeds, in this study we genotyped almost half of the extant cattle populations of Reggiana and Modenese breeds. We then used genome‐wide information in comparative analyses to detect signatures of selection that might be derived by the divergent directional selection and genetic drifts that have contributed to shape the genome structures of these two autochthonous cattle breeds.

## 2 MATERIALS AND METHODS

### 2.1 Ethic statement

Animal samples used in this study were collected following the recommendation of directive 2010/632.1.

### 2.2 Animals and genotyping data

A total of 1435 cattle included in this study (Reggiana, *n* = 1109; Modenese, *n* = 326) were genotyped with the GeneSeek GGP Bovine 150k SNP chip following the manufacturer's protocol. PLINK software v. 1.9 (Chang et al., 2015) was used to filter genotyping data. Only markers with minor allele frequency (MAF) <0.01 across the two breeds, with a call rate >90% in each breed, mapped in unique positions in the autosomes of the ARS‐UCD1.2 cattle genome version were retained.

The Herd Book of the Reggiana breed (ANABORARE, 2020) considers that the Reggiana cattle should have the homozygous recessive genotype *e/e* at the *MC1R* gene, which causes the red coat colour of the breed (Bovo et al., 2021; Klungland et al., 1995; Russo et al., 2007). For this reason, further filtering was applied, retaining for this study only Reggiana cattle with the *e/e* genotype that could be retrieved from the GeneSeek GGP Bovine 150 k SNP chip.

After filtering, the data set accounted for 1109 Reggiana samples (98 samples were excluded because they were heterozygous *E/e* at the *MC1R* gene and 2 samples did not pass the quality criteria for genotyping), 326 Modenese samples and a total of 128,574 markers.

### 2.3 Population genomic analyses

Observed and expected heterozygosity (H_O_ and H_E_, respectively) were calculated with PLINK v. 1.9 (Chang et al., 2015). Inbreeding coefficient of an individual (I) relative to the subpopulation (S) (F_IS_), fixation index (F_ST_) and inbreeding coefficient of an individual (I) relative to the total (T) population (F_IT_) were calculated with VCFtools software (Danecek et al., 2011).

Linkage disequilibrium (LD) was measured using *r^2^* for all SNP pairs of each chromosome using PLINK v. 1.9 (Chang et al., 2015) and within‐breed LD decay was estimated using bins of 10 kb. Plots were generated in R v.3.5.1. (R Core Team, 2018) with the ggplot2 package (Wickam, 2016). Recent and historical effective population size (Ne) were estimated using the SNeP software (Barbato et al., 2015), using the maximum distance between SNP to be analysed of 10 Mb and the binwidth of 100 kb for the calculation of LD.

To perform population structure analyses, pruning of SNPs in high LD was carried out of PLINK 1.9 (Chang et al., 2015) with the‐‐indep‐pairwise command (options: window size of 50 kb, step size of 10 and *r^2^* threshold of 0.1). A total of 14,131 SNPs was retained (average of 487 ± 166 SNPs for each chromosome). Multidimensional scaling (MDS) analysis was performed using a matrix of genome‐wide identity‐by‐state (IBS) pairwise distances as implemented in PLINK 1.9 (Chang et al., 2015).

Population stratification was evaluated with the ADMIXTURE v3.1 software (Alexander et al., 2009). Analyses were preformed considering the number of subpopulations (K) that ranged from 1 to 39 and retaining the cross‐validation error (CV) for each K.

### 2.4 F_ST_ analyses, gene annotation and haploblock analysis

The fixation index F_ST_ is a measure of population genetic differentiation. F_ST_ provides information on the genomic variation at a locus between populations relative to that within populations (Wright, 1951). F_ST_ is based on the measure of differences in allele frequencies between populations, which capture loci that are differentially fixed as results of differential selection. High F_ST_ values indicate local positive selection, which is a characteristic of genomic regions that have gone through differential selection, whereas low F_ST_ values suggest negative or neutral selection. Therefore, F_ST_ is particularly suitable to detect signatures of selection between breeds as it is less affected by genetic drift than other methods proposed to detect signatures of selection across livestock breeds (e.g. Zhao et al., 2015).

Wright's F_ST_ for each SNP in the pairwise comparison between Reggiana and Modenese populations was calculated with PLINK 1.9 (Chang et al., 2015) using the method of Weir and Cockerham (1984). Overall averaged F_ST_ was calculated considering all SNPs in the pairwise comparisons.

Signatures of selection were determined using pairwise F_ST_ analyses using two approaches: (i) single‐marker pairwise F_ST_ analysis and (ii) averaged genome window F_ST_ comparative analysis. The SNP with the top 0.0005% F_ST_ (99.95^th^ percentile; top 64 SNPs) defined the threshold to detect signatures of selection. In the window‐based approach, 4,987 windows of 1 Mb with a step of 500 kb, were tested by computing an average F_ST_ based of SNPs overlapping the window. A total of 9,953 windows of 500 kb with a step of 250 kb were also tested. Analyses, based on method of Weir and Cockerham (1984) were performed with VCFtools (Danecek et al., 2011). All windows that contained at least four SNPs were then retained. The top mean F_ST_ (mF_ST_) 20 windows were considered in these analyses (99.6^th^ and 99.8^th^ percentiles for the 1‐Mb and 0.5‐Mb window‐based analyses, respectively).

Annotation of the genome regions including the SNPs and windows that trespassed the defined threshold was retrieved from the *Bos taurus* genome assembly version ARS‐UCD1.2 and considering a region of ± 200 kb around the detected SNPs or considering the overlapping or partially overlapping windows. Genes were retrieved using Ensembl Biomart tool (http://www.ensembl.org/biomart/martview/) and then evaluated considering their functional roles according to an extensive literature search.

Functional gene enrichment analysis of genes closed to the SNPs detected in the single‐marker F_ST_ analysis was performed with Enrichr (Chen et al., 2013). Over‐representation analysis run over the Gene Ontology v.2021 (http://geneontology.org), KEGG v.2021 (http://www.kegg.jp/) and GWAS catalogue v.2019 (https://www.ebi.ac.uk/gwas/) human libraries. Terms with an adjusted *p*‐value <0.05 and at least two input genes were retained as statistically over‐represented.

Haplotype block analysis of the *MC1R* and *ASIP* gene regions was performed using the software Haploview v. 4.2 (Barret et al., 2005) using default options.

## 3 RESULTS

### 3.1 Population genomic parameters and structures of the two cattle breeds

Table 1 summarises some basic population genomic parameters calculated in the two cattle breeds. The average within‐breed MAF was higher in the Reggiana (mean and standard deviation: 0.271 ± 0.147) breed than in the Modenese breed (0.257 ± 0.151). The MAF distribution (Figure S1) confirmed the highest number of SNPs (*n* = 8085) with the lowest MAF values (ranging from 0.01 to 0.05) that was detected in the Modenese breed than in Reggiana (*n* = 6,649). Within‐breed H_O_ and H_E_ heterozygosity were lower in Modenese than in Reggiana (Table 1) reflecting the other SNP‐based information reported above.

**TABLE 1:** Population genomic parameters calculated in the Reggiana and Modenese cattle breeds

Breed	No. of animals	Average MAF^1^	H_O_^2^	H_E_^3^	F_IS_^4^	F_IT_^5^
Reggiana	1109	0.271 ( ± 0.147)	0.359	0.360	0.003	0.068
Modenese	326	0.257 ( ± 0.151)	0.354	0.349	−0.017	0.050

^1^Minor allele frequency and standard deviation in brackets.

^2^Observed heterozygosity.

^3^Expected heterozygosity.

^4^Inbreeding coefficient of an individual (I) relative to the subpopulation (S).

^5^Inbreeding coefficient of an individual (I) relative to the total (T) population.

**FIGURE 2:**
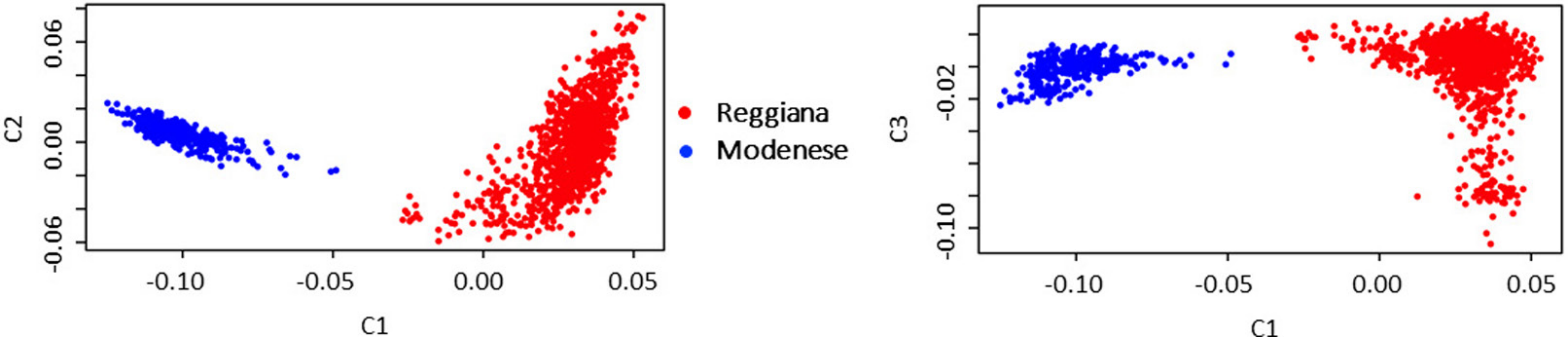
Multidimensional scaling plots produced using genotyping information from each investigated cattle of the Reggiana (red dots) and Modenese (blue dots) breeds. Different components (C) are considered in the plots [Colour figure can be viewed at wileyonlinelibrary.com]

Figure 2 reports the two‐dimensional MDS‐plots obtained using genome information from the Reggiana and Modenese breeds. The two breeds are separated by the first three coordinates into two compact and distinguishable clouds.

**FIGURE 3:**
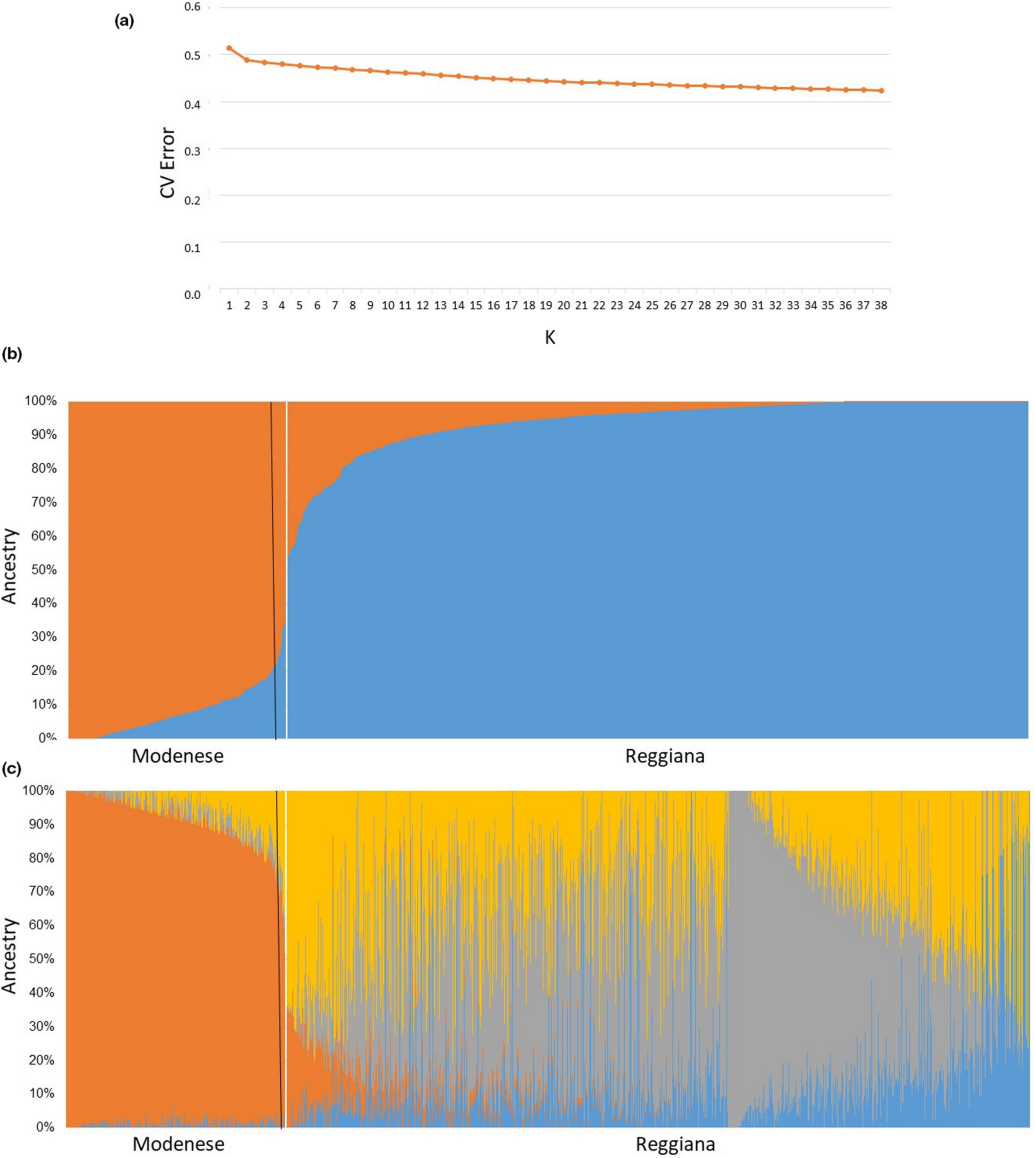
Results of the ADMIXTURE analysis. a) Cross validation (CV) error with K from 1 to 39. b) Plot distribution with K = 2. c) Plot distribution with K = 4. For the last two plots, putative subpopulations (therefore, 2 for K = 2 and 4 for K = 4) are labelled with a different colour [Colour figure can be viewed at wileyonlinelibrary.com]

The results of the ADMIXTURE analysis are showed in Figure 3. Despite a high number of K was considered, the minimum value of CV error was not detected. However, the higher decrease in K is observed with K = 2, and after that the K values remains quite constant (Figure 3a). The population stratification at K = 2 (Figure 3b) is consistent with the clusters detected by the MDS analysis. If a higher K is considered (e.g. K = 4; Figure 3c), a higher level of stratification of the Reggiana breed could be observed in contrast with the Modenese breed that tended to be more homogeneous.

**FIGURE 4:**
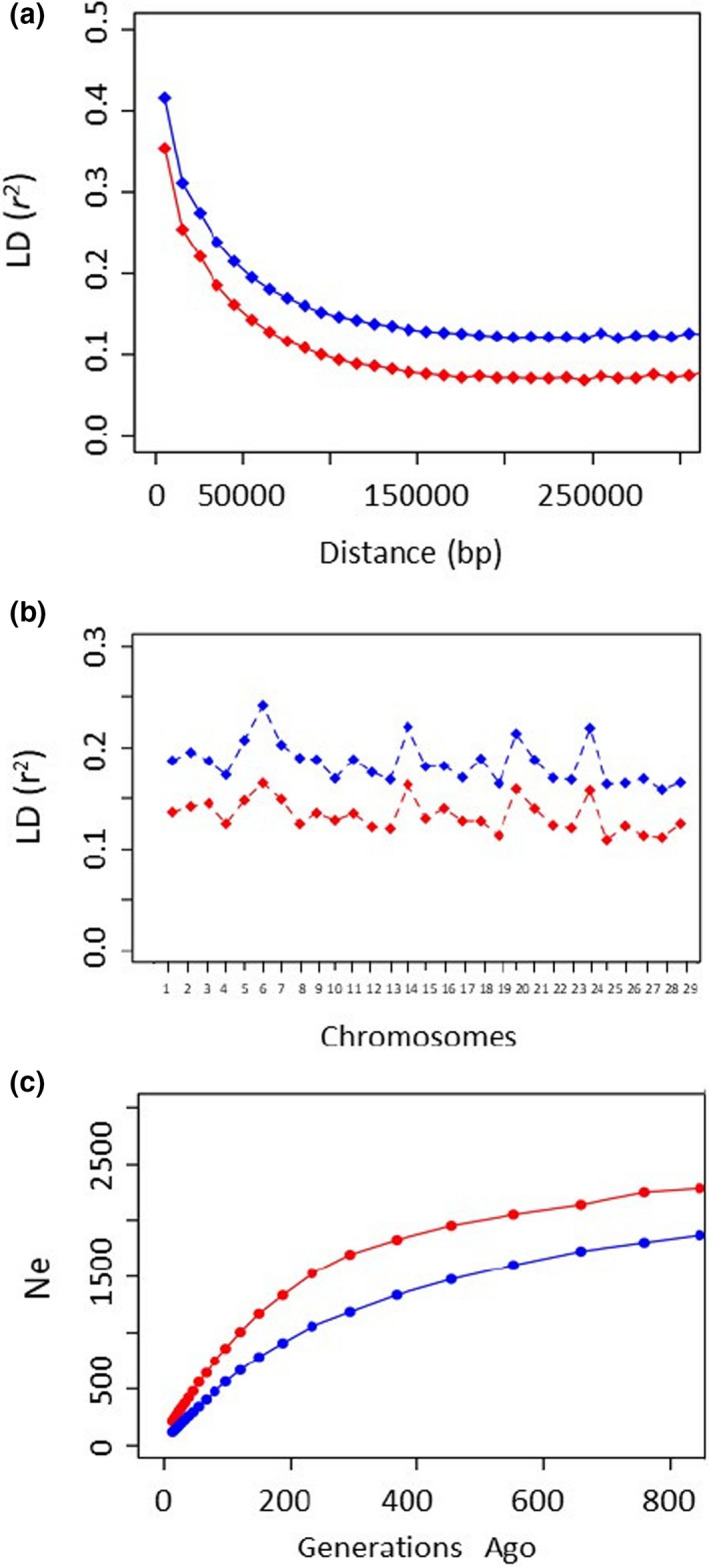
Population genomic parameters represented in the Reggiana (red points and lines) and in Modenese (blue points and lines) breeds: (a) linkage disequilibrium (LD) decay over distance; (b) average LD calculated for all autosomes; (c) effective population size (Ne) over the past generations [Colour figure can be viewed at wileyonlinelibrary.com]

Linkage disequilibrium was higher in the Modenese breed than in the Reggiana breed as it was evident from the LD decay (Figure 4a) and the average LD calculated for all autosomes in the two breeds (Figure 4b and Table S1). This information reflected the lower Ne value obtained in the Modenese breed than in the Reggiana breed (120 vs. 215, respectively) as also evidenced from its progressive decline plot over the past generations (Figure 4c). The similar trend in LD values estimated for each chromosome in the two breeds, which confirmed the general higher LD values in the Modenese than in the Reggiana breed, also evidenced that the SNPs present in the chip might largely affect the LD structure in the two cattle breeds.

### 3.2 F_ST_ derived signatures of selection between the two breeds

**FIGURE 5:**
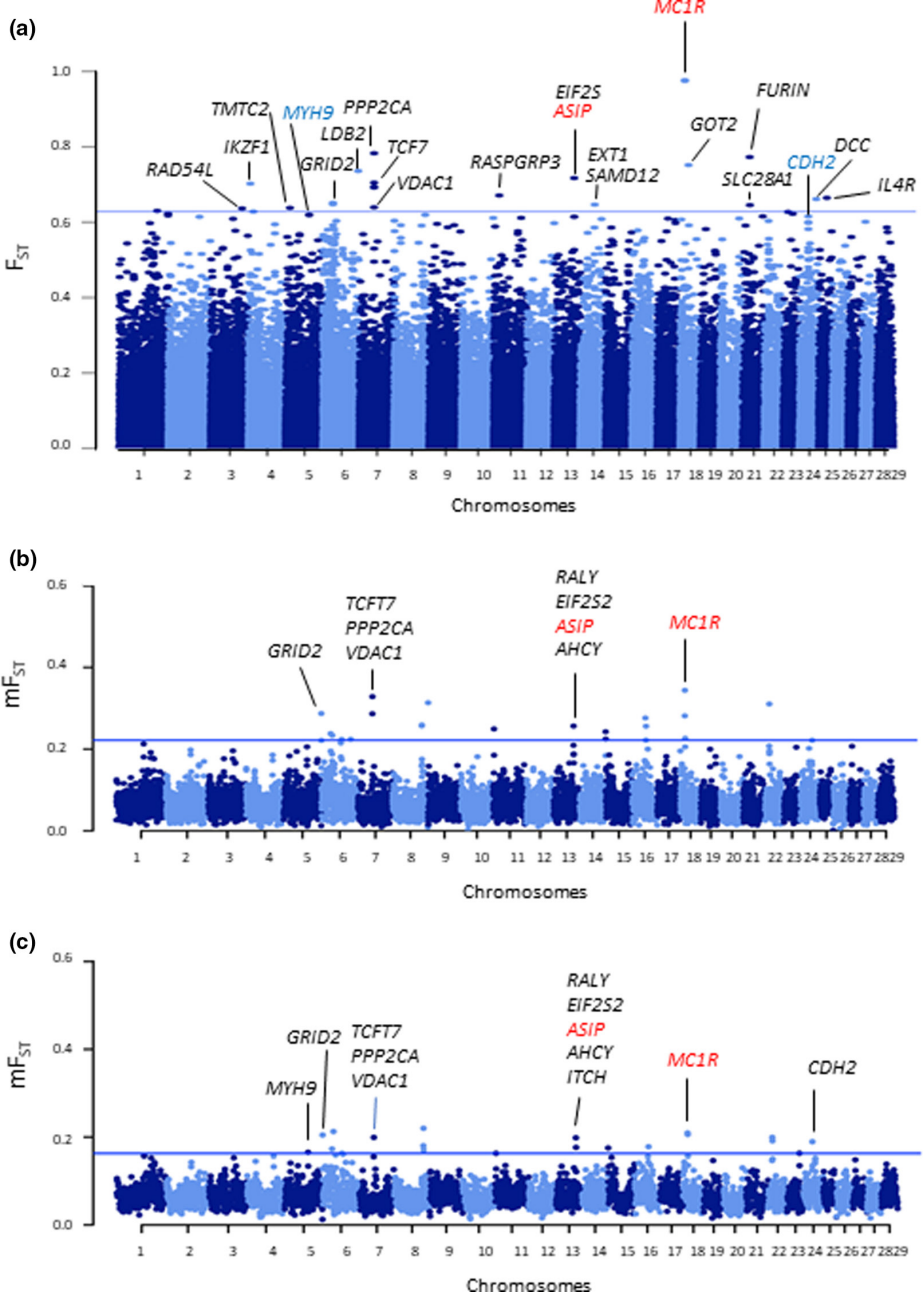
Manhattan plots obtained in the single‐marker (a) and window‐based F_ST_ analyses using windows of 0.5 Mb (b) or windows of 1 Mb (c), in which the y axis reports the mean F_ST_ values (mF_ST_). In the single‐marker analysis, the top 20 markers have been annotated, including two markers within the top 64 list, which are close to genes (in blue) that have been also contained in windows detected with the window‐based approaches. The regions detected with the window‐based approaches (b and c) are annotated with the genes close to SNPs reported in the single‐marker analysis. The two main coat colour genes are indicated in red. A few genes in the *ASIP* region on BTA13 identified in the window‐based analyses are annotated. The threshold lines are defined according to the percentiles reported in Materials and methods [Colour figure can be viewed at wileyonlinelibrary.com]

The global averaged F_ST_ value across all SNPs obtained comparing Reggiana and Modenese was 0.066. Figure 5 reports the Manhattan plots obtained in the single‐marker (a) and window‐based (b and c) F_ST_ analyses that compared genomic information of the Reggiana and Modenese breeds.

Table 2 reports the top 20 markers with the highest F_ST_ values, which ranged from 0.977 to 0.637. The full set of markers (*n* = 64) trespassing the 99.95^th^ percentile threshold is reported in Table S2. Table 3 includes the top 20 0.5 Mb genome windows and Table S3 contains information on the top 20 1 Mb genome windows identified using the two applied window‐based analyses, respectively. Averaged F_ST_ values in these windows ranged from 0.344 to 0.222 in the top (99.8^th^ percentile) 0.5‐Mb genome windows and from 0.255 to 0.163 in the top (99.6^th^ percentile) 1‐Mb genome windows. The drastic drop of F_ST_ values from the single‐marker to the window‐based analyses might indicate that the two breeds could be distinguished by a few highly separated loci that experienced a rapid decay of LD apart from informative short genome regions, diluting the F_ST_ values in the window‐based approaches.

**TABLE 2:** Top 20 markers identified in the single‐marker F_ST_ analysis between the two breeds

Markers^1^	BTA^2^	Position^3^	F_ST_	Closest gene (bp)^4^
MC1R	18	14,705,645	0.977	*MC1R* (0)*
BovineHD0700013748	7	45,833,400	0.783	*PPP2CA* (9618)*
ARS‐BFGL‐NGS−114140	21	21,791,054	0.773	*FURIN* (0)
ARS‐BFGL‐NGS−28154	18	26,500,840	0.752	*GOT2* (53,100)
BovineHD0600033381	6	112,511,216	0.736	*LDB2* (357,854)
BovineHD1300018297	13	63,480,254	0.717	*EIF2S2* (0), *ASIP* (182,542)*
BTA−78954‐no‐rs	7	45,800,275	0.705	*TCF7* (0)*
ARS‐BFGL‐NGS−55059	4	5,545,419	0.702	*IKZF1* (0)
ARS‐BFGL‐NGS−5595	7	45,766,695	0.694	*TCF7* (2218)*
ARS‐BFGL‐NGS−73679	7	45,729,837	0.692	*TCF7* (39,076)*
BTA−86548‐no‐rs	11	16,591,322	0.671	*RASPGRP3* (476,598)
BovineHD2500007120	25	24,908,014	0.665	*IL4R* (0)
BovineHD2400015179	24	53,014,583	0.661	*DCC* (0)
BovineHD0600009128	6	31,158,986	0.652	*GRID2* (0)*
BovineHD0600009122	6	31,135,482	0.647	*GRID2* (0)*
ARS‐BFGL‐NGS−35081	14	46,102,133	0.647	*SAMD12* (36,424)*, EXT1* (62,323)
BovineHD2100006752	21	22,531,247	0.645	*SLC28A1* (0)
ARS‐BFGL‐NGS−20141	7	45,691,037	0.639	*VDAC1* (0)*
BovineHD0500003920	5	12,981,358	0.638	*TMTC2* (405,765)
ARS‐BFGL‐NGS−16203	3	99,840,480	0.637	*RAD54L* (0)

Note

The markers are ranked according to the F_ST_ value. All 99.95^th^ percentile markers are reported in Table S2.

^1^Marker name in the GeneSeek GGP Bovine 150 k SNP chip.

^2^ *Bos taurus* chromosome.

^3^Position of the marker in the ARS‐UCD1.2 cattle genome version.

^4^Distance in bp of the marker with the indicated gene is reported within the bracket. When the marker overlaps the gene, a value equal to 0 bp is indicated. The star symbol indicates those genes that are also included in the top 0.5 and/or 1 Mb windows in the window‐based F_ST_ analyses (see also Figure 5).

**TABLE 3:** The top 20 0.5 Mb genome windows identified in the F_ST_ analysis between the two breeds

BTA^1^	Bin start^2^	Bin end^3^	No. of SNPs^4^	Average F_ST_^5^	Genes^6^
18	14,500,001	15,000,000	18	0.344	*CPNE7, DPEP1, CHMP1A, CDK10, SPATA2L, VPS9D1, ZNF276, FANCA, SPIRE2, TCF25, MC1R, TUBB3, DEF8, DBNDD1, GAS8, U1, SHCBP1, VPS35*
7	45,750,001	46,250,000	15	0.328	*TCF7, SKP1, PPP2CA, CDKL3, UBE2B, CDKN2AIPNL, JADE2, SAR1B, U6, SEC24A, CAMLG*
22	13,250,001	13,750,000	15	0.310	*ENTPD3, RPL14, ZNF619, ZNF621, 7SK, U6*
6	1	500,000	19	0.287	*U6, APELA*
7	45,500,001	46,000,000	19	0.286	*C7H5orf15, VDAC1, TCF7, SKP1, PPP2CA, CDKL3, UBE2B, CDKN2AIPNL*
18	14,250,001	14,750,000	17	0.281	*CDH15, SLC22A31, ANKRD11, SPG7, RPL13, CPNE7, DPEP1, CHMP1A, CDK10, SPATA2L, VPS9D1, ZNF276, FANCA, SPIRE2, TCF25, MC1R, TUBB3, DEF8*
16	42,250,001	42,750,000	10	0.276	*UBIAD1, MTOR, ANGPTL7, EXOSC10, SRM, MASP2, TARDBP*
8	93,250,001	93,750,000	21	0.259	*SMC2*
8	93,000,001	93,500,000	23	0.257	*‐*
13	63,250,001	63,750,000	21	0.257	*CHMP4B, PXMP4, E2F1, ZNF341, NECAB3, RALY, EIF2S2, ASIP, AHCY*
16	44,250,001	44,750,000	6	0.256	*U1, GPR157, CA6, ENO1, U6*
11	2,750,001	3,250,000	28	0.249	*CNNM4, CNNM3, ANKRD23, ANKRD39, SEMA4C, COX5B, ACTR1B*
15	1	500,000	22	0.243	*–*
6	30,000,001	30,500,000	22	0.238	*PDLIM5, 7SK, HPGDS, SMARCAD1*
6	35,000,001	35,500,000	28	0.234	*SNCA*
18	15,500,001	16,000,000	22	0.227	*NETO2, ITFG1, U6, PHKB*
15	250,001	750,000	23	0.225	*–*
6	94,250,001	94,750,000	15	0.224	*U6, ANTXR2*
6	65,250,001	65,750,000	30	0.224	*COX7B2, H4C14, GABRA4, GABRB1*
16	44,000,001	44,500,000	13	0.222	*SPSB1, H6PD, U1, GPR157, CA6*

Note

The windows are ranked according to the average F_ST_ value.

^1^ *Bos taurus* chromosome.

^2^Start position of the genome window in the ARS‐UCD1.2 cattle genome version.

^3^End position of the genome window in the ARS‐UCD1.2 cattle genome version.

^4^Number of SNPs included in the 0.5 Mb genome window.

^5^Average F_ST_ value based on SNPs included in the genome window.

^6^Genes annotated in the reported genome window (ARS‐UCD1.2 cattle genome version).

The 64 markers were distributed in 23 different autosomes (Table S2). Some of them were also captured in the window‐based analyses: eight markers encompassing three chromosomes overlapped the top 0.5‐Mb windows and 15 markers encompassing five chromosomes overlapped the top 1‐Mb windows (Table S2). The top marker (F_ST_ = 0.977) was the frameshift mutation in the *MC1R* gene that causes the *e* allele at the *Extension* locus and that determines the classical red coat colour of the Reggiana cattle (Bovo et al., 2021; Klungland et al., 1995; Russo et al., 2007). This result was due to the fact that Reggiana cattle selected for this study were fixed for this recessive *MC1R* allele whereas Modenese cattle were almost fixed for an alternative allele (only one Modenese animal carried the *e* allele). This polymorphic site, located on BTA18, was in the first top genome window of the 0.5 Mb analysis and in the third and fourth top sliding windows in the 1 Mb analysis. The haploblock structure of this region in Reggiana cattle indicated that a relatively low level of LD is present in the *MC1R* gene region in both Reggiana and Modenese breeds, with some LD blocks only upstream or downstream this gene (Figure S2).

The second top polymorphism in the single‐marker analysis was localized on BTA7 about 9.6 kb from the *protein phosphatase 2 catalytic subunit alpha* (*PPP2CA*) gene (Table 2). Additional four markers in the same chromosome region (Table 2), that contributed to the second and fifth highest averaged F_ST_ values in the 0.5‐Mb genome window analysis (Table 3), were within or close to the *transcription factor 7* (*TCF7*) and *voltage dependent anion channel 1* (*VDAC1*) genes.

Other three top 99.95^th^ percentile markers, that were also contained in top genome windows considering both the 0.5‐Mb and 1‐Mb size, were located on BTA13 within or very close to the *eukaryotic translation initiation factor 2 subunit beta* (*EIF2S2*) and *agouti signalling protein* (A*SIP*) genes (Table 2 and Table 3; Table S2). *ASIP* is the gene that determines the *Agouti* locus, which affects coat colour in many mammalian species (Searle, 1968). The LD structure of this region in Modenese cattle showed a haplotype block in the correspondence of the *RALY heterogeneous nuclear ribonucleoprotein* (*RALY*) and *eukaryotic translation initiation factor 2 subunit beta* (*EIF2S2*) genes, which are upstream the *ASIP* gene (Figure S3). The Reggiana breed had a main haploblock in the correspondence of the *itchy E3 ubiquitin protein ligase* (*ITCH*) gene, which is downstream the *ASIP* gene (Figure S3).

Additional top SNPs on BTA5, BTA6 and BTA24 were also included in top genome windows detected with the 0.5 and/or 1 Mb window‐approaches. Markers on BTA5 were within or close to the *myosin heavy chain 9* (*MYH9*) gene, SNPs on BTA6 were within the *glutamate ionotropic receptor delta type subunit 2* (*GRID2*) gene and the marker on BTA24 was in a desert region where the closest genes are *cadherin 2* (*CDH2*) and *cadherin related 23* (*CDH23*) (Table 3, Table S2 and Table S3).

Gene enrichment analysis returned significant results only for the Human GWAS catalogue. Amongst the 12 over‐represented phenotypes (Table S4), nine were related to pigmentation (e.g. skin colour, skin pigmentation, skin ageing, freckles and tanning processes) and involved the *ASIP*, *ERBB4*, *EIF2S2* and *MC1R* genes. Melanogenesis was the most over‐represented KEGG pathway (adjusted *p‐*value = 0.14; *ASIP*, *MC1R* and *TCF7* genes) whereas the regulation of tyrosine phosphorylation of STAT protein (GO:0,042,509; adjusted *p‐*value = 0.15; *ERBB4*, *GHR* and *PPP2CA* genes) was the most over‐represented GO Biological Process.

## 4 DISCUSSION

Reggiana and Modenese are considered two iconic breeds that are part of the history of the livestock production sector of the North of Italy from which the well‐known PDO Parmigiano‐Reggiano cheese was originated. Genomic population parameters calculated for the two breeds are in agreement to those that usually describe the small population size of many autochthonous breeds.

The geographical closeness of the two breeds (both were developed in close provinces of the North of Italy), as it could be expected, also resulted in a relatively high genetic closeness when Reggiana and Modenese were analysed together with many other Italian cattle breeds (Mastrangelo, Sardina, et al., 2018). Despite this closeness, Reggiana and Modenese cattle were clearly distinguished using genomic information obtained with the GeneSeek GGP Bovine 150k SNP chip. Admixture patterns and MDS‐plots were able to separate all animals belonging to the two breeds. Genomic differences could be derived by the combined action of divergent artificial directional selection and genetic drift followed by genetic isolation due to the use of different male and female genetic stocks. A pairwise comparison between these two breeds, shown in Figure 5, and the subsequent genetic investigation highlighted that the most relevant genomic differences may affect coat colour genes. Gene enrichment analysis confirmed that pigmentation and related traits explained the most relevant differences that emerged between these two breeds. This is of particular interest as these genomic differences might be eventually masked or under‐rated if comparisons would have included more breeds in averaged F_ST_ analyses, which are the commonly used methodologies for these types of investigations (Munoz et al., 2019; Bovo et al., 2020).

Both Reggiana and Modenese breeds originally derived from unspecialized triple purpose cattle (work‐dairy‐beef); therefore, one of the main drivers that contributed to separate them and that represents the main characterizing phenotype is their different coat colour. Solid red (Reggiana) and solid white with grey shades (Modenese) are the colours that define the standards of these two breeds. This phenotype is the most relevant descriptor used to admit animals in one or the other breed herd book. There is a story telling tradition that suggests that the selection for different coat colours in Reggiana and Modenese would derive from the ancient rivalry between the two close towns (i.e. Reggio Emilia and Modena) from which the two breeds took their names.

The most relevant signature of selection that differentiated Reggiana and Modenese breeds was determined at the *e* allele of the *MC1R* gene, which causes the classical red (fromentino) coat colour of the Reggiana cattle (Bovo et al., 2021; Klungland et al., 1995; Russo et al., 2007). The high F_ST_ value (almost equal to 1) reached by the causative mutation at the *MC1R* allele dropped in the window‐based analyses, as it was averaged across all SNPs included in 0.5 Mb or 1 Mb. The relatively low LD that is present in the *MC1R* gene region of BTA18 in the Reggiana indicated an ancient origin of the fixed *e* allele in this breed and that more haplotypes or haplotype blocks containing this causative mutation were present in Reggiana cattle. The strong selection pressure that fixed (or almost fixed) this *Extension* allele, therefore, did not result in an extended fixation of several other close SNPs on BTA18.

Instead, the genetic determinism of the white coat colour with some pale grey shades of the Modenese cattle, has not been unravelled yet. This type of coat colour, also described in several other taurine breeds, have not been fully clarified in any other populations as well. Modenese breed is almost fixed for the wild type allele at the *MC1R* gene, as also reported in a previous study (Russo et al., 2007). According to the classical epistatic interaction between the *Extension* and *Agouti* loci, wild type alleles at the *MC1R* gene would give the possibility to express mutated alleles at the *Agouti* locus (Searle, 1968). Therefore, it is quite remarkable that a strong F_ST_ signal between Reggiana and Modenese was detected in the *ASIP* gene region on BTA13. The signal was not as high as it was observed for the *MC1R* gene region even if it was confirmed using single‐marker and the two window‐based approaches. The relatively lower F_ST_ value of this region if compared to that of the *MC1R* gene region could be due to the lack of the causative mutation(s) in the SNP chip and/or to the masking effect of the mutated *MC1R* allele in Reggiana that would epistatically cover the mutated allele(s) at the *ASIP* gene. Mutated *ASIP* alleles could be also present in the Reggiana breed but at lower frequency than in Modenese breed, reducing in this way allele frequency differences between the two breeds and, in turn the F_ST_ values in this BTA13 region. The LD analysis in Reggiana and Modenese indicated high LD in different regions of the BTA13 that includes *ASIP*, suggesting the presence of different haplotype structures in the two breeds, with different potential regulatory effects over *ASIP* that we could hypothesize (which should be, however, demonstrated).

Variability at the *ASIP* gene determined by copy number variations (CNVs), probably with regulatory effects on gene expression, has been already associated with the white coat colour in different sheep and goat breeds (Norris & Whan, 2008; Fontanesi et al., 2009, 2011). Therefore, it would be possible to speculate that, in Modenese breed, CNVs or other regulatory mutations affecting *ASIP* gene could determine a similar phenotypic effect on coat colour as already observed in the other two ruminant species (i.e. sheep and goat). Recently, Trigo et al. (2021) reported that in Nellore cattle (*Bos indicus*), which are selected for white coat colour, a structural variant affecting the *ASIP* gene expression is associated with darker coat pigmentation on specific parts of the body. Few studies have investigated variability in the *Bos taurus ASIP* gene. Royo et al (2005) did not report any variability in the *ASIP* coding region of cattle from six Spanish and three French brown breeds. None of the *ASIP* polymorphisms reported in Korean cattle were associated to any coat colours (Do et al., 2007). Girardot et al. (2006) reported in Normande cattle an insertion in a regulatory region of the *ASIP* gene that was suggested to be implicated in the brindle coat colour pattern of the breed. It will be important to characterize the *ASIP* gene in Modenese cattle, including all regulatory regions and surrounding genes, to disentangle its expected effect on coat colour that it could be possible to predict from the results of this study.

To further support the hypothesised role of the *ASIP* gene (or of close genes on BTA13) in determining the white coat colour of the Modenese cattle, no signatures of selection were determined in genomic regions harbouring other genes affecting coat colour in cattle. This would exclude the involvement of other well‐known genes determining white patterns, like the *v‐kit Hardy‐Zuckerman 4 feline sarcoma viral oncogene homolog* (*KIT*) gene on BTA6 and the *microphthalmia‐associated transcription factor* (*MITF*) gene on BTA22 (Fontanesi, Scotti, et al., 2010; Fontanesi, Tazzoli, et al., 2012, 2010), or genes diluting coat colours, like the *premelanosome protein* (*PMEL*) gene on BTA5, which was recently shown to dilute the coat colour in Reggiana cattle (Bovo et al., 2021).

Other signatures of selection were evident from the F_ST_ comparative analyses between the two breeds. These signatures might be caused by genetic drift that would be subsequently due to the constrains generated by the use of sires and dams that could assure the requested coat colour phenotype needed to register the animals to their herd books. These genomic differences could contribute to further differentiate these two breeds for some production performances or other phenotypic traits, but their effect should be demonstrated using other approaches. The use of other pairwise methods to detect signature of selection (e.g. Bertolini et al., 2020) could also identify additional genomic regions that might be involved in differentiating these breeds or that could provide more complete genomic pictures of the results of the genetic drift, bottleneck and admixture with other breeds or populations that have probably contributed to shape the current genetic pool of these two cattle autochthonous breeds.

## 5 CONCLUSIONS

Population genomic analyses applied to compare the genome architecture of two closely related cattle genetic resources (Reggiana and Modenese) allow the identification of hints that could explain their main phenotypic differences. Signatures of selection were evidenced in two genome regions encompassing major coat colour‐affecting genes. One region on BTA18, including the *MC1R* gene, whose role in determining the red coat colour of Reggiana was already well established, could provide a proof of concept for the general interpretation of the results obtained in a region of BTA13, which includes the *ASIP* gene. Whole genome resequencing of Reggiana and Modenese is currently planned. This will help to characterise variability in the *ASIP* gene and investigate their association with the white coat colour in Modenese breed. The identification of Modenese specific DNA markers could be useful to develop methods to authenticate the origin of the milk and thus of the cheese declared to be derived from this breed.

This study demonstrates how population genomic approaches designed to take advantage from the diversity between local genetic resources could provide interesting information to explain exterior traits not yet completely investigated in cattle.

## CONFLICT OF INTEREST

The authors declare they do not have any competing interests. Data reported in this work can be shared after signature of an agreement on their use with University of Bologna.

## AUTHOR CONTRIBUTION

L.F. designed the study, interpreted the results and obtained funding. L.F., F.B. and G.M. wrote the paper. A.R. and G.M. performed the wet lab work. G.M., F.B., G.S., S.B. and M. Ba. conducted bioinformatic analyses. M. Bo. and M.P. provided samples and data. S.B., G.S., S.D. and M. Ba. contributed to data interpretation. All authors read and approved the submitted version.

## Supporting information

Supplementary Material

## Data Availability

Data reported in this work can be shared after signature of an agreement on their use with University of Bologna.

## References

[jbg12659-bib-0001] Alexander, D. H. , Novembre, J. , & Lange, K. (2009). Fast model‐based estimation of ancestry in unrelated individuals. Genome Research, 19, 1655–1664. 10.1101/gr.094052.109 19648217 PMC2752134

[jbg12659-bib-0002] ANABORARE . (2009). Retieved from https://www.razzareggiana.it/

[jbg12659-bib-0003] Barbato, M. , Orozco‐terWengel, P. , Tapio, M. , & Bruford, M. W. (2015). SNeP: A tool to estimate trends in recent effective population size trajectories using genome‐wide SNP data. Frontiers in Genetics, 6, 109. 10.3389/fgene.2015.00109 25852748 PMC4367434

[jbg12659-bib-0004] Barrett, J. C. , Fry, B. , Maller, J. , & Daly, M. J. (2005). Haploview: Analysis and visualization of LD and haplotype maps. Bioinformatics, 21, 263–265. 10.1093/bioinformatics/bth457 15297300

[jbg12659-bib-0005] Bertolini, F. , Galimberti, G. , Calò, D. G. , Schiavo, G. , Matassino, D. , & Fontanesi, L. (2015). Combined use of principal component analysis and random forests identify population‐informative single nucleotide polymorphisms: Application in cattle breeds. Journal of Animal Breeding and Genetics, 132, 346–356. 10.1111/jbg.12155 25781205

[jbg12659-bib-0006] Bertolini, F. , Galimberti, G. , Schiavo, G. , Mastrangelo, S. , Di Gerlando, R. , Strillacci, M. G. , Bagnato, A. , Portolano, B. , & Fontanesi, L. (2018). Preselection statistics and Random Forest classification identify population informative single nucleotide polymorphisms in cosmopolitan and autochthonous cattle breeds. Animal, 12, 12–19. 10.1017/S1751731117001355 28643617

[jbg12659-bib-0007] Bertolini, F. , Schiavo, G. , Bovo, S. , Sardina, M. T. , Mastrangelo, S. , Dall'Olio, S. , Portolano, B. , & Fontanesi, L. (2020). Comparative selection signature analyses identify genomic footprints in Reggiana cattle, the traditional breed of the Parmigiano‐Reggiano cheese production system. Animal, 14, 921–932. 10.1017/S1751731119003318 31928542

[jbg12659-bib-0008] Bovo, S. , Ribani, A. , Muñoz, M. , Alves, E. , Araujo, J. P. , Bozzi, R. , Čandek‐Potokar, M. , Charneca, R. , Di Palma, F. , Etherington, G. , Fernandez, A. I. , García, F. , García‐Casco, J. , Karolyi, D. , Gallo, M. , Margeta, M. , Martins, M. J. , Mercat, M. J. , Moscatelli, G. , … Fontanesi, L. (2020). Whole‐genome sequencing of European autochthonous and commercial pig breeds allows the detection of signatures of selection for adaptation of genetic resources to different breeding and production systems. Genetics Selection Evolution, 52, 33. 10.1186/s12711-020-00553-7 PMC7318759 32591011

[jbg12659-bib-0009] Bovo, S. , Schiavo, G. , Kazemi, H. , Moscatelli, G. , Ribani, A. , Ballan, M. , Bonacini, M. , Prandi, M. , Dall'Olio, S. , & Fontanesi, L. (2021). Exploiting within‐breed variability in the autochthonous Reggiana breed identified several candidate genes affecting pigmentation‐related traits, stature and udder defects in cattle. Animal Genetics, 52, 579–597. 10.1111/age.13109 34182594 PMC8519023

[jbg12659-bib-0010] Catillo, G. , Moioli, B. , Napolitano, F. , & Steri, R. (2018). Identification of genomic regions harboring diversity between Holstein and two local endangered breeds, Modenese and Maremmana. Livestock Science, 216, 75–83. 10.1016/j.livsci.2018.07.011

[jbg12659-bib-0011] Chang, C. C. , Chow, C. C. , Tellier, L. C. , Vattikuti, S. , Purcell, S. M. , & Lee, J. J. (2015). Second‐generation PLINK: Rising to the challenge of larger and richer datasets. Gigascience, 4, 7. 10.1186/s13742-015-0047-8 25722852 PMC4342193

[jbg12659-bib-0012] Chen, E. Y. , Tan, C. M. , Kou, Y. , Duan, Q. , Wang, Z. , Meirelles, G. V. , Clark, N. R. , & Ma'ayan, A. (2013). Enrichr: Interactive and collaborative HTML5 gene list enrichment analysis tool. BMC Bioinformatics, 14, 128. 10.1186/1471-2105-14-128 23586463 PMC3637064

[jbg12659-bib-0013] Danecek, P. , Auton, A. , Abecasis, G. , Albers, C. A. , Banks, E. , DePristo, M. A. , Handsaker, R. E. , Lunter, G. , Marth, G. T. , Sherry, S. T. , McVean, G. , & Durbin, R. (2011). 1000 Genomes project analysis group. The variant call format and VCFtools. Bioinformatics, 27, 2156–2158. 10.1093/bioinformatics/btr330 21653522 PMC3137218

[jbg12659-bib-0014] Do, K. T. , Shin, H. Y. , Lee, J. H. , Kim, N. S. , Park, E. W. , Yoon, D. H. , & Kim, K. S. (2007). Investigation of coat color candidate genes in Korean cattle (Hanwoo). Journal of Animal Science and Technology, 49, 711–718. 10.3390/d6040705

[jbg12659-bib-0015] Felius, M. , Beerling, M. L. , Buchanan, D. S. , Theunissen, B. , Koolmees, P. A. , & Lenstra, J. A. (2014). On the history of cattle genetic resources. Diversity, 6, 705–750. 10.3390/d6040705

[jbg12659-bib-0016] Fontanesi, L. (2009). Genetic authentication and traceability of food products of animal origin: New developments and perspectives. Italian Journal of Animal Science, 8, 9–18. 10.4081/ijas.2009.s2.9

[jbg12659-bib-0017] Fontanesi, L. , Beretti, F. , Riggio, V. , González, E. G. , Dall'Olio, S. , Davoli, R. , Russo, V. , & Portolano, B. (2009). Copy number variation and missense mutations of the agouti signaling protein (ASIP) gene in goat breeds with different coat colors. Cytogenetic and Genome Research, 126, 333–347. 10.1159/000268089 20016133

[jbg12659-bib-0018] Fontanesi, L. , Dall’Olio, S. , Beretti, F. , Portolano, B. , & Russo, V. (2011). Coat colours in the Massese sheep breed are associated with mutations in the agouti signalling protein (ASIP) and melanocortin 1 receptor (MC1R) genes. Animal, 5, 8–17. 10.1017/S1751731110001382 22440696

[jbg12659-bib-0019] Fontanesi, L. , Scotti, E. , & Russo, V. (2010). Analysis of SNPs in the KIT gene of cattle with different coat colour patterns and perspectives for using these markers for breed traceability and authentication of beef and dairy products. Italian Journal of Animal Science, 9, e42. 10.4081/ijas.2010.e42

[jbg12659-bib-0020] Fontanesi, L. , Scotti, E. , & Russo, V. (2012). Haplotype variability in the bovine MITF gene and association with piebaldism in holstein and simmental cattle breeds. Animal Genetics, 43, 250–256. 10.1111/j.1365-2052.2011.02242.x 22486495

[jbg12659-bib-0021] Fontanesi, L. , Scotti, E. , Samorè, A. B. , Bagnato, A. , & Russo, V. (2015). Association of 20 candidate gene markers with milk production and composition traits in sires of Reggiana breed, a local dairy cattle population. Livestock Science, 176, 14–21. 10.3168/jds.2017-12666

[jbg12659-bib-0022] Fontanesi, L. , Scotti, E. , Tazzoli, M. , Beretti, F. , Dall’Olio, S. , Davoli, R. , & Russo, V. (2007). Investigation of allele frequencies of the growth hormone receptor (GHR) F279Y mutation in dairy and dual purpose cattle breeds. Italian Journal of Animal Science, 6, 415–420. 10.4081/ijas.2007.415

[jbg12659-bib-0023] Fontanesi, L. , Tazzoli, M. , Russo, V. , & Beever, J. (2010). Genetic heterogeneity at the bovine KIT gene in cattle breeds carrying different putative alleles at the spotting locus. Animal Genetics, 41, 295–303. 10.1111/j.1365-2052.2009.02007.x 19968642

[jbg12659-bib-0024] Gandini, G. , Maltecca, C. , Pizzi, F. , Bagnato, A. , & Rizzi, R. (2007). Comparing local and commercial breeds on functional traits and profitability: The case of Reggiana dairy cattle. Journal of Dairy Science, 90, 2004–2011. 10.3168/jds.2006-204 17369242

[jbg12659-bib-0025] Girardot, M. , Guibert, S. , Laforet, M. P. , Gallard, Y. , Larroque, H. , & Oulmouden, A. (2006). The insertion of a full‐length Bos taurus LINE element is responsible for a transcriptional deregulation of the Normande Agouti gene. Pigment Cell Research, 19, 346–355. 10.1111/j.1600-0749.2006.00312.x 16827753

[jbg12659-bib-0026] Klungland, H. , Vage, D. I. , Gomez‐Raya, L. , Adalsteinsson, S. , & Lien, S. (1995). The role of melanocyte‐stimulating hormone (MSH) receptor in bovine coat color determination. Mammalian Genome, 6, 636–639. 10.1007/BF00352371 8535072

[jbg12659-bib-0027] Mastrangelo, S. , Ciani, E. , Ajmone‐Marsan, P. , Bagnato, A. , Battaglini, L. , Bozzi, R. , Carta, A. , Catillo, G. , Cassandro, M. , Casu, S. , Ciampolini, R. , Crepaldi, P. , D’Andrea, M. , Di Gerlando, R. , Fontanesi, L. , Longeri, M. , Macciotta, N. P. P. , Mantovani, R. , Marletta, D. , … Pilla, F. (2018). Conservation status and historical relatedness of Italian cattle breeds. Genetics Selection Evolution, 50, 35. 10.1186/s12711-018-0406-x PMC6019226 29940848

[jbg12659-bib-0028] Mastrangelo, S. , Sardina, M. T. , Tolone, M. , Di Gerlando, R. , Sutera, A. M. , Fontanesi, L. , & Portolano, B. (2018). Genome‐wide identification of runs of homozygosity islands and associated genes in local dairy cattle breeds. Animal, 12, 2480–2488. 10.1017/S1751731118000629 29576040

[jbg12659-bib-0029] Mastrangelo, S. , Tolone, M. , Di Gerlando, R. , Fontanesi, L. , Sardina, M. T. , & Portolano, B. (2016). Genomic inbreeding estimation in small populations: Evaluation of runs of homozygosity in three local dairy cattle breeds. Animal, 10, 746–754. 10.1017/S1751731115002943 27076405

[jbg12659-bib-0030] Muñoz, M. , Bozzi, R. , García‐Casco, J. , Núñez, Y. , Ribani, A. , Franci, O. , García, F. , Skrlep, M. , Schiavo, G. , Bovo, S. , Utzeri, V. J. , Charneca, R. , Martins, J. M. , Quintanilla, R. , Tibau, J. , Margeta, V. , Djurkin‐Kusec, I. , Mercat, M. J. , Riquet, J. , … Ovilo, C. (2019). Genomic diversity, linkage disequilibrium and selection signatures in European local pig breeds assessed with a high density SNP chip. Scientific Reports, 9, 13464. 10.1038/s41598-019-49830-6 31537860 PMC6753209

[jbg12659-bib-0031] Norris, B. J. , & Whan, V. A. (2008). A gene duplication affecting expression of the ovine ASIP gene is responsible for white and black sheep. Genome Research, 18, 1282–1293. 10.1101/gr.072090.107 18493018 PMC2493430

[jbg12659-bib-0032] Petrera, F. , Catillo, G. , Napolitano, F. , Malacarne, M. , Franceschi, P. , Summer, A. , & Abeni, F. (2016). New insights into the quality characteristics of milk from Modenese breed compared with Italian Friesian. Italian Journal of Animal Science, 15, 559–567. 10.1080/1828051X.2016.1222889

[jbg12659-bib-0033] R Core Team (2018). R: A language and environment for statistical computing. R Foundation for Statistical Computing. https://www.R‐project.org/org/

[jbg12659-bib-0034] Royo, L. J. , Alvarez, I. , Fernández, I. , Arranz, J. J. , Gómez, E. , & Goyache, F. (2005). The coding sequence of the *ASIP* gene is identical in nine wild‐type coloured cattle breeds. Journal of Animal Breeding and Genetics, 122, 357–360. 10.1111/j.1439-0388.2005.00541.x 16191045

[jbg12659-bib-0035] Russo, V. , Fontanesi, L. , Scotti, E. , Tazzoli, M. , Dall’Olio, S. , & Davoli, R. (2007). Analysis of melanocortin 1 receptor (MC1R) gene polymorphisms in some cattle breeds their usefulness and application for breed traceability and authentication of Parmigiano Reggiano cheese. Italian Journal of Animal Science, 6, 257–272. 10.4081/ijas.2007.257

[jbg12659-bib-0036] Scotti, E. , Fontanesi, L. , Schiavini, F. , La Mattina, V. , Bagnato, A. , & Russo, V. (2010). DGAT1 p. K232A polymorphism in dairy and dual purpose Italian cattle breeds. Italian Journal of Animal Science, 9, e16. 10.4081/ijas.2010.e16

[jbg12659-bib-0037] Searle, A. G. (1968). Comparative genetics of coat colour in mammals. Logos Press.

[jbg12659-bib-0038] Trigo, B. B. , Utsunomiya, A. T. H. , Fortunato, A. A. A. D. , Milanesi, M. , Torrecilha, R. B. P. , Lamb, H. , Nguyen, L. , Ross, E. M. , Hayes, B. , Padula, R. C. M. , Sussai, T. S. , Zavarez, L. B. , Cipriano, R. S. , Caminhas, M. M. T. , Lopes, F. L. , Pelle, C. , Leeb, T. , Bannasch, D. , Bickhart, D. , … Utsunomiya, Y. T. (2021). Variants at the ASIP locus contribute to coat color darkening in Nellore cattle. Genetics Selection Evolution, 53, 40. 10.1186/s12711-021-00633-2 PMC8082809 33910501

[jbg12659-bib-0039] Weir, B. S. , & Cockerham, C. C. (1984). Estimating F‐statistics for the analysis of population structure. Evolution, 38, 1358–1370. 10.2307/2408641 28563791

[jbg12659-bib-0040] Wickham, H. (2016). ggplot2: Elegant graphics for data analysis. Springer‐Verlag. https://ggplot2.tidyverse.org. ISBN 978‐3‐319‐24277‐4.

[jbg12659-bib-0041] Wright, S. (1951). The genetical structure of populations. Annals of Eugenics, 15, 323–354. 10.1111/j.1469-1809.1949.tb02451.x 24540312

[jbg12659-bib-0042] Zhao, F. , McParland, S. , Kearney, F. , Du, L. , & Berry, D. P. (2015). Detection of selection signatures in dairy and beef cattle using high‐density genomic information. Genetics Selection Evolution, 47, 49. 10.1186/s12711-015-0127-3 PMC4472243 26089079

