# Supplementary material for: Signatures of selection are present in the genome of two close autochthonous cattle breeds raised in the North of Italy and mainly distinguished for their coat colours
Source: J Anim Breed Genet. 2021 Nov 28;139(3):307–19. doi: 10.1111/jbg.12659 (PMC9300179; doi:10.1111/jbg.12659)
Supplement: Supplementary file 1 — Supplementary Material [file JBG-139-307-s001.docx]

**Supplementary material**

Signatures of selection are present in the genome of two close autochthonous cattle breeds raised in the North of Italy and mainly distinguished for their coat colours

Francesca Bertolini, Giulia Moscatelli, Giuseppina Schiavo, Samuele Bovo, Anisa Ribani, Mohamad Ballan, Massimo Bonacini, Marco Prandi, Stefania Dall’Olio, Luca Fontanesi

**Figure S1.** Distribution of the different classes of minor allele frequencies (MAF) for the markers genotyped in the Reggiana and Modenese breeds.
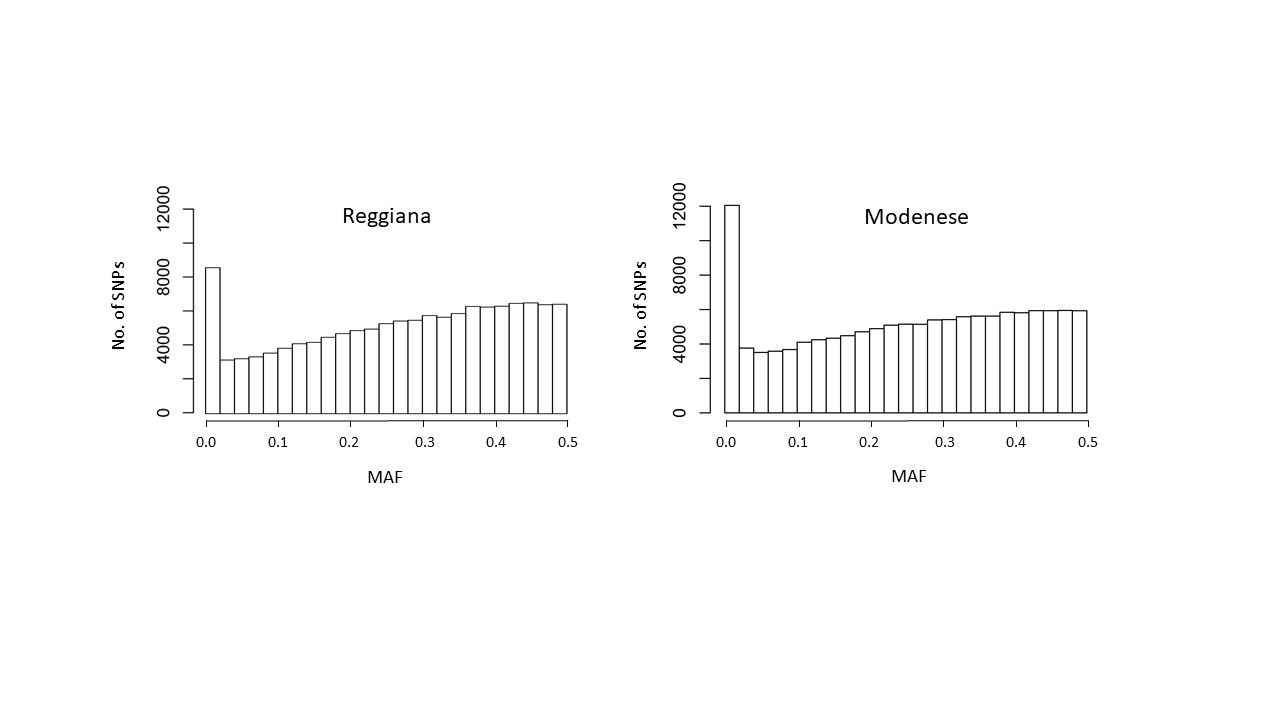


**Figure S2.** Linkage disequilibrium (*r*^2^) plot of the chromosome 18 region encompassing the *MC1R* gene, obtained for the Reggiana and Modenese breeds. The genes annotated in this region are reported at the top of the two haploblocks.


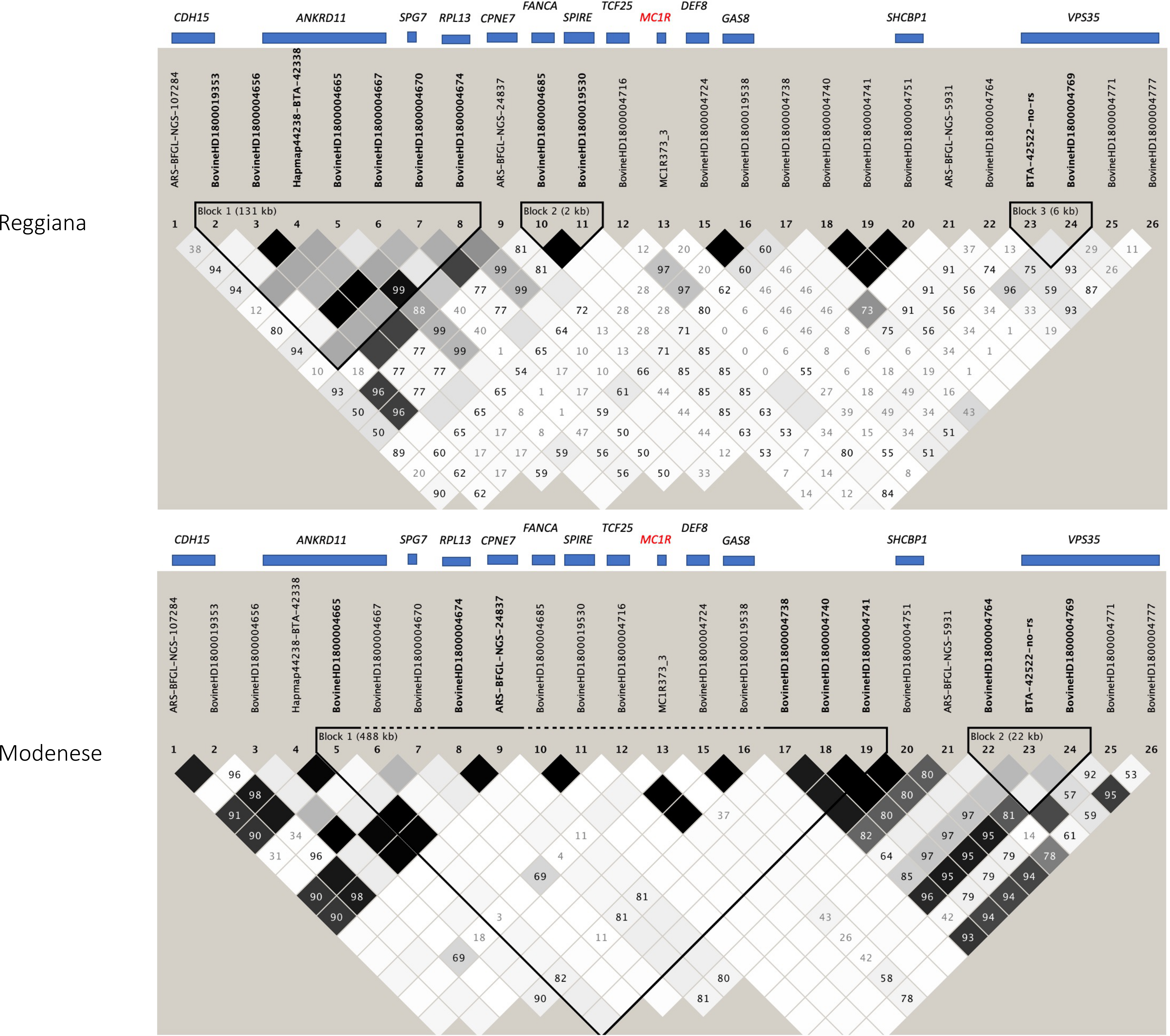


**Figure S3.** Linkage disequilibrium (*r*^2^) plot of the chromosome 13 region encompassing the *ASIP* gene, obtained for the Reggiana and Modenese breeds. The genes annotated in this region are reported at the top of the two haploblocks.


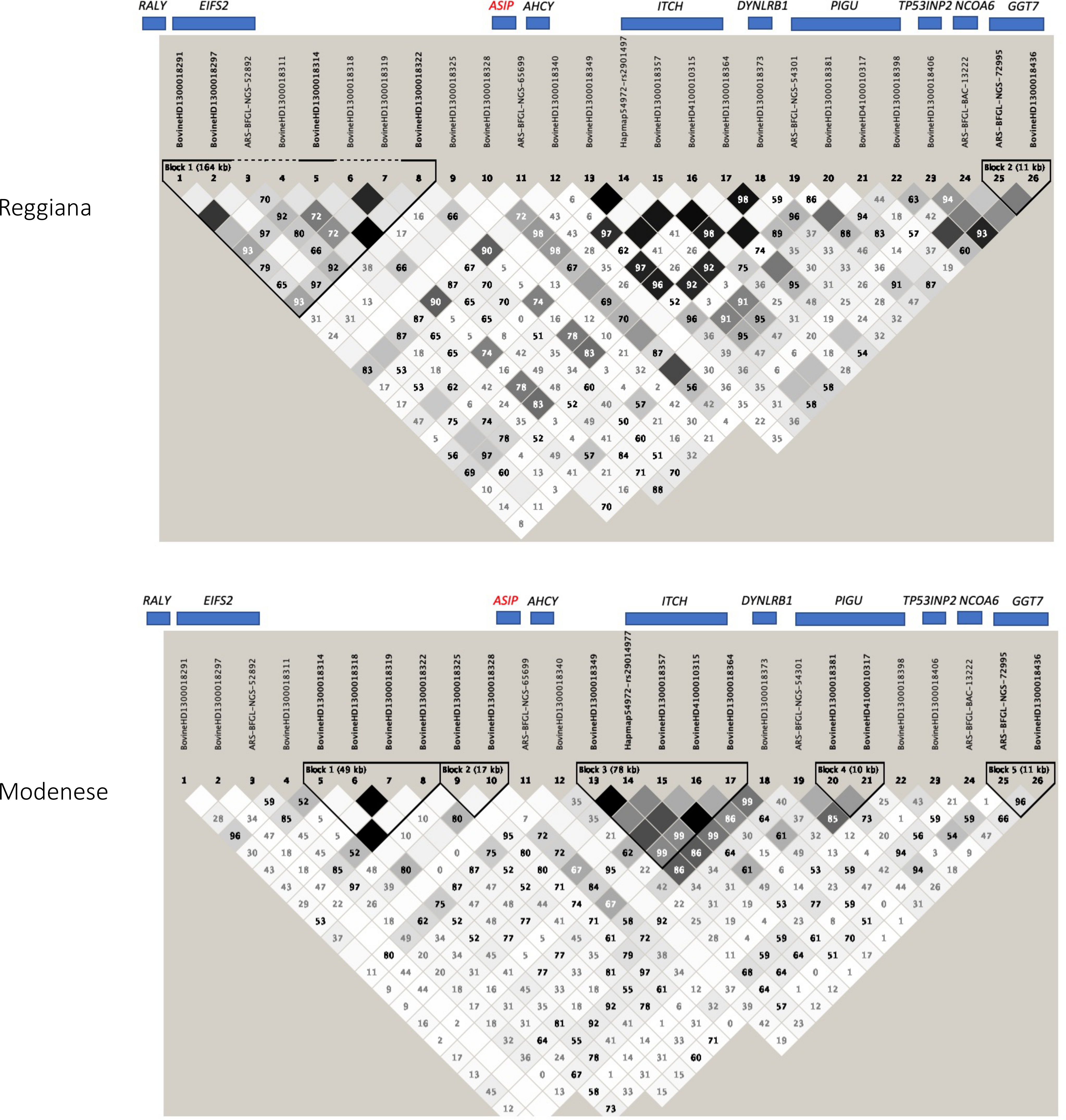


**Table S1.** The average linkage disequilibrium value (*r*^2^) for the markers in all autosomes reported for the Reggiana and Modenese breeds.

| **BTA^1^** | ***r*^2^ Reggiana (s.d.)** | ***r*^2^ Modenese (s.d.)** |
| --- | --- | --- |
| 1 | 0.136 (0.205) | 0.187 (0.243) |
| 2 | 0.142 (0.210) | 0.195 (0.250) |
| 3 | 0.145 (0.222) | 0.187 (0.247) |
| 4 | 0.125 (0.199) | 0.173 (0.228) |
| 5 | 0.148 (0.228) | 0.207 (0.267) |
| 6 | 0.165 (0.248) | 0.241 (0.296) |
| 7 | 0.149 (0.227) | 0.203 (0.261) |
| 8 | 0.125 (0.195) | 0.190 (0.244) |
| 9 | 0.135 (0.210) | 0.188 (0.244) |
| 10 | 0.128 (0.193) | 0.169 (0.222) |
| 11 | 0.135 (0.203) | 0.188 (0.246) |
| 12 | 0.122 (0.192) | 0.175 (0.231) |
| 13 | 0.120 (0.191) | 0.168 (0.225) |
| 14 | 0.163 (0.250) | 0.220 (0.287) |
| 15 | 0.130 (0.204) | 0.181 (0.236) |
| 16 | 0.140 (0.212) | 0.181 (0.237) |
| 17 | 0.128 (0.196) | 0.170 (0.225) |
| 18 | 0.128 (0.199) | 0.189 (0.241) |
| 19 | 0.114 (0.180) | 0.164 (0.217) |
| 20 | 0.159 (0.244) | 0.213 (0.273) |
| 21 | 0.140 (0.210) | 0.188 (0.245) |
| 22 | 0.123 (0.189) | 0.170 (0.226) |
| 23 | 0.121 (0.197) | 0.169 (0.231) |
| 24 | 0.157 (0.240) | 0.219 (0.302) |
| 25 | 0.109 (0.178) | 0.164 (0.218) |
| 26 | 0.123 (0.187) | 0.165 (0.219) |
| 27 | 0.113 (0.181) | 0.169 (0.223) |
| 28 | 0.111 (0.175) | 0.158 (0.209) |
| 29 | 0.125 (0.189) | 0.165 (0.221) |

^1^ *Bos taurus* chromosome.

**Table S2.** All markers with Fst values above the 99.8^th^ percentile distribution identified in the single-marker F_ST_ analysis between the two breeds.

| **Markers^1^** | **BTA^2^** | **Position^3^** | **F_ST_** | **Closest gene (bp)^4^** |
| --- | --- | --- | --- | --- |
| MC1R*^ | 18 | 14705645 | 0.977 | *MC1R* (0) |
| BovineHD0700013748*^ | 7 | 45833400 | 0.783 | *PPP2CA* (9618) |
| ARS-BFGL-NGS-114140 | 21 | 21791054 | 0.773 | *FURIN* (0) |
| ARS-BFGL-NGS-28154 | 18 | 26500840 | 0.752 | *GOT2* (53100) |
| BovineHD0600033381 | 6 | 112511216 | 0.736 | *LDB2* (357854) |
| BovineHD1300018297*^ | 13 | 63480254 | 0.717 | *EIF2S2* (0), *ASIP* (182542) |
| BTA-78954-no-rs*^ | 7 | 45800275 | 0.705 | *TCF7* (0) |
| ARS-BFGL-NGS-55059 | 4 | 5545419 | 0.702 | *IKZF1* (0) |
| ARS-BFGL-NGS-5595*^ | 7 | 45766695 | 0.694 | *TCF7* (2218) |
| ARS-BFGL-NGS-73679*^ | 7 | 45729837 | 0.692 | *TCF7* (39076) |
| BTA-86548-no-rs | 11 | 16591322 | 0.671 | *RASPGRP3* (476598) |
| BovineHD2500007120 | 25 | 24908014 | 0.665 | *IL4R* (0) |
| BovineHD2400015179 | 24 | 53014583 | 0.661 | *DCC* (0) |
| BovineHD0600009128 | 6 | 31158986 | 0.652 | *GRID2* (0) |
| BovineHD0600009122 | 6 | 31135482 | 0.647 | *GRID2* (0) |
| ARS-BFGL-NGS-35081 | 14 | 46102133 | 0.647 | *SAMD12* (36424)*, EXT1* (62323) |
| BovineHD2100006752 | 21 | 22531247 | 0.645 | *SLC28A1* (0) |
| ARS-BFGL-NGS-20141*^ | 7 | 45691037 | 0.639 | *VDAC1*(0) |
| BovineHD0500003920 | 5 | 12981358 | 0.638 | *TMTC2* (405765) |
| ARS-BFGL-NGS-16203 | 3 | 99840480 | 0.637 | *RAD54L* (0) |
| Hapmap55173-rs29024142 | 1 | 121487166 | 0.630 | *PLSCR5* (365630) |
| Hapmap50265-BTA-13206 | 4 | 15203507 | 0.628 | *ASNS* (0) |
| ARS-BFGL-NGS-25325 | 23 | 14974356 | 0.627 | *APOBEC2* (0) |
| BovineHD0100046358 | 1 | 156520533 | 0.622 | *KCNH8* (0) |
| BTA-94041-no-rs | 23 | 29663663 | 0.622 | *ORP2* (0) |
| BovineHD0500021352^ | 5 | 74767531 | 0.620 | *MYH9* (0) |
| BovineHD0500021349^ | 5 | 74753566 | 0.620 | *MYH9* (0) |
| BovineHD0500021332^ | 5 | 74715894 | 0.620 | *MYH9* (0) |
| BTB-00370549 | 8 | 99625705 | 0.619 | *PALM2* (96246) |
| ARS-BFGL-NGS-40570^ | 5 | 74717288 | 0.618 | *MYH9* (0) |
| BovineHD0100046348 | 1 | 156507162 | 0.617 | *KCNH8* (0) |
| BovineHD2400007601^ | 24 | 27560729 | 0.616 | *-* |
| Hapmap24484-BTA-136154 | 21 | 63972179 | 0.616 | *BCL11B* (221357) |
| BovineHD1300018322*^ | 13 | 63629244 | 0.615 | *ASIP* (33552) |
| BovineHD0200028587 | 2 | 98950479 | 0.614 | *ERBB4* (273296) |
| BovineHD1300018314*^ | 13 | 63579877 | 0.614 | *ASIP* (82919) |
| ARS-BFGL-NGS-43715 | 11 | 86912838 | 0.612 | *ATP6V1C2* (0) |
| BovineHD1000029475 | 10 | 100564643 | 0.611 | *TTC8* (2400) |
| BovineHD2000016914 | 20 | 60190001 | 0.610 | *-* |
| BTB-01307961 | 17 | 33562176 | 0.609 | *ANKRD50* (393929) |
| BovineHD0300020926 | 3 | 70918424 | 0.609 | *LRRIQ* (200093) |
| ARS-BFGL-BAC-20448 | 14 | 67928364 | 0.608 | *PTDSS1* (0) |
| BovineHD1800000963 | 18 | 3286986 | 0.607 | *CNTNAP4* (133529) |
| BovineHD1700016954 | 17 | 57518753 | 0.606 | *KSR2* (0) |
| BovineHD0800000070 | 8 | 503066 | 0.604 | *ANXA10* (0) |
| BovineHD0600011794 | 6 | 41996260 | 0.604 | *ADGRA3* (0) |
| BovineHD2200003649 | 22 | 12417613 | 0.603 | *WDR48* (0) |
| Hapmap41322-BTA-64648 | 28 | 9215236 | 0.601 | *HEATR1* (0) |
| BovineHD2400007580 | 24 | 27449561 | 0.600 | *-* |
| BovineHD4100016540^ | 24 | 27570575 | 0.598 | *CDH23* (133529) |
| ARS-BFGL-NGS-112404 | 17 | 57495561 | 0.598 | *KSR2* (0) |
| BovineHD0100028276 | 1 | 98305974 | 0.597 | *MECOM* (0) |
| BovineHD1200026502 | 12 | 86367927 | 0.597 | *ATP11A* (0) |
| BTB-01073083 | 9 | 24493304 | 0.595 | *RASPO3* (306182) |
| BovineHD0400023583 | 4 | 84535781 | 0.594 | *KCND2* (132580) |
| BovineHD4100004382 | 6 | 31590806 | 0.592 | *GRID2* (50650) |
| BovineHD0900022313 | 9 | 79439745 | 0.592 | *NMBR* (138468) |
| BovineHD0800006437 | 8 | 20952293 | 0.592 | *-* |
| BovineHD1800008609 | 18 | 27586155 | 0.592 | *-* |
| BovineHD2100001969 | 21 | 8498932 | 0.591 | *ARRDC4* (235849) |
| BovineHD0800026512 | 8 | 87886263 | 0.591 | *-* |
| BovineHD1100001176 | 11 | 3287227 | 0.589 | *TMEM131* (0) |
| BovineHD0900016418 | 9 | 58935377 | 0.586 | *-* |
| BovineHD2900009743 | 29 | 32109387 | 0.586 | *GHR* (0) |

^1^ Marker name in the GeneSeek GGP Bovine 150k SNP chip. Markers overlapping with the window-based 0.5 Mb and 1 Mb is reported with the “*” and “^” symbols, respectively.

^2^ *Bos taurus* chromosome.

^3^ Position, in base pairs, of the marker in the ARS-UCD1.2 cattle genome version.

^4^ Distance in base pairs of the marker with the indicated gene is reported within the brackets. When the marker overlaps the gene, a distance equal to 0 bp is indicated.

**Table S3.** Top 1 Mb genome windows identified in the F_ST_ analysis between the two breeds. The windows are ranked according to the average F_ST_ value.

| **BTA^1^** | **Bin start^2^** | **Bin end^3^** | **No. of SNPs^4^** | **Average F_ST_^5^** | **Genes^6^** |
| --- | --- | --- | --- | --- | --- |
| 8 | 93000001 | 94000000 | 42 | 0.255 | *SMC2* |
| 6 | 34500001 | 35500000 | 51 | 0.213 | *CCSER1, MMRN1, SNCA* |
| 18 | 14000001 | 15000000 | 39 | 0.209 | *CDT1, APRT, GALNS, TRAPPC2L, CBFA2T3, ACSF3, CDH15, SLC22A31, ANKRD11* |
| 18 | 14500001 | 15500000 | 37 | 0.206 | *CPNE7, DPEP1, CHMP1A, CDK10, SPATA2L, VPS9D1, ZNF276, FANCA, SPIRE2, TCF25, MC1R, TUBB3, DEF8, DBNDD1, GAS8, U1, SHCBP1, VPS35, ORC6, MYLK3, C18H16orf87, GPT2, DNAJA2, NETO2* |
| 6 | 1 | 1000000 | 50 | 0.205 | *APELA* |
| 22 | 13000001 | 14000000 | 41 | 0.199 | *EIF1B, ENTPD3, ZNF619, RPL14, ZNF621, CTNNB1, ULK4* |
| 7 | 45500001 | 46500000 | 41 | 0.199 | *C7H5orf15, VDAC1, TCFT7, SKP1, PPP2CA, CDKL3, UBE2B, CDKN2AIPNL, JADE2, SAR18, SEC24A, CAMLG, DDX46, PCBD2, TXNDC15, C7H5orf24, CATSPER3, PITX1* |
| 13 | 63000001 | 64000000 | 38 | 0.198 | *C13H20orf144, CHMP4B, PXMP4, E2F1, ZNF341, NECAB3, RALY, EIF2S2, ASIP, AHCY, ITCH, DUNLRB1, PIGU, MP1LC3A, NCOA6, TP53INP2* |
| 22 | 13500001 | 14500000 | 46 | 0.192 | *CTNNB1, ULK4, TRAK1, CCK* |
| 24 | 27500001 | 28500000 | 77 | 0.189 | *CDH2* |
| 8 | 92500001 | 93500000 | 49 | 0.180 | *CYLC2* |
| 16 | 44000001 | 45000000 | 18 | 0.178 | *SPSB1, H6PD, GPR157, CA6, ENO1, RERE, SLC45A1* |
| 13 | 63500001 | 64500000 | 35 | 0.176 | *ASIP, AHCY, ITCH, DYNLRB1, MAP1LC3A, PIGU, TP53INP2, NCOA6, GGT7, ACSS2, GSS, MYH7B, bta-mir-499, TRPC4AP, EDEM2, PROCR* |
| 15 | 1 | 1000000 | 41 | 0.175 | *-* |
| 6 | 30000001 | 31000000 | 49 | 0.173 | *PDLIM5, 7SK, Metazoa_SRP, HPGDS, SMARCAD1, ATOH1, GRID2* |
| 8 | 93500001 | 94500000 | 31 | 0.171 | *SMC2, OR13C3, OR13C8* |
| 5 | 74000001 | 75000000 | 52 | 0.165 | *RBFOX2, APOL3, MYH9, U6, TXN2, FOXRED2, EIF3D, CACNG2* |
| 23 | 38500001 | 39500000 | 64 | 0.163 | *5S_rRNA, RNF144B, DEK, KDM1B, 5S_rRNA, TPMT, NHLRC1, U6* |
| 6 | 62000001 | 63000000 | 45 | 0.163 | *KCTD8* |
| 11 | 2500001 | 3500000 | 65 | 0.163 | *NEURL3, KANSL3, ARID5A, U6, FER1L5, LMAN2L, CNNM4, CNNM3, ANKRD23, ANKRD39, SEMA4C, COX5B, ACTR1B, ZAP70, TMEM131, VWA3B* |

^1^ *Bos taurus* chromosome.

^2^ Start position, in base pairs,of the genome window in the ARS-UCD1.2 cattle genome version.

^3^ End position, in base pairs, of the genome window in the ARS-UCD1.2 cattle genome version.

^4^ Number of single nucleotide polymorphisms (SNPs) included in the 1 Mb genome window.

^5^ Average F_ST_ value based on the SNPs included in the genome window.

^6^ Genes annotated in the reported genome window (ARS-UCD1.2 cattle genome version).

**Table S4.** Results of the gene enrichment analysis. Significant results were obtained only with the Human GWAS catalog. The most significant KEGG pathways and GO Biological Processes are also reported.

| **Library** | **Term** | **Overlap^1^** | ***p*-value^2^** | **Genes^3^** |
| --- | --- | --- | --- | --- |
| GWAS catalog | Skin sensitivity to sun | 2/6 | 0.016 | *MC1R*, *ASIP* |
|  | Facial pigmentation | 2/7 | 0.016 | *MC1R*, *ASIP* |
|  | Skin colour saturation | 2/9 | 0.017 | *MC1R*, *ASIP* |
|  | Tanning | 2/11 | 0.017 | *MC1R*, *ASIP* |
|  | Freckles | 2/12 | 0.017 | *MC1R*, *ASIP* |
|  | Skin aging (microtopography measurement) | 2/13 | 0.017 | *MC1R*, *ERBB4* |
|  | Skin pigmentation | 2/13 | 0.017 | *ASIP*, *EIF2S2* |
|  | Non-melanoma skin cancer | 2/23 | 0.040 | *MC1R*, *ASIP* |
|  | Post bronchodilator FEV1/FVC ratio | 4/206 | 0.040 | *GRID2*, *PALM2*, *HEATR1*, *KSR2* |
|  | Low tan response | 2/24 | 0.040 | *MC1R*, *ASIP* |
|  | Feeling fed-up | 2/29 | 0.049 | *ERBB4*, *DCC* |
|  | Monoclonal gammopathy of undetermined significance | 2/29 | 0.049 | *ERBB4*, *KSR2* |
| KEGG pathways* | Melanogenesis | 3/101 | 0.137 | *MC1R*, *TCF7*, *ASIP* |
| GO Biological Process* | Regulation of tyrosine phosphorylation of STAT protein (GO:0042509) | 3/68 | 0.147 | *PPP2CA*, *GHR*, *ERBB4* |

^1^ Number of genes of the input set over the number of genes annotated with the term.

^2^ Adjusted *p*-value.

^3^ Genes of the input set annotated with the term.

* Not statistically valid (adjusted *p*-value > 0.05).
